# *Qip* gene in *Fusarium oxysporum* is required for normal hyphae morphology and virulence

**DOI:** 10.1080/21501203.2015.1027313

**Published:** 2015-03-25

**Authors:** Lin Cheng, Jian Ling, Liqin Liang, Zhongqin Luo, Jie Zhang, Bingyan Xie

**Affiliations:** a College of Life Science, Shanxi Normal University, Gong yuan Street No. 1, Yaodu, Linfen041004, China; b Institute of Vegetables and Flowers, Chinese Academy of Agricultural Sciences, South Street No. 12, Zhongguancun, Haidian, Beijing100081, China

**Keywords:** RNA-silencing mechanism, *Fusarium oxysporum*, *Qip* gene, homologous recombination technology, hyphal growth and development, pathogenicity

## Abstract

Ribonucleic acid (RNA)-silencing mechanisms exist in many eukaryotes to regulate a variety of biological processes. The known molecular components are related to Dicers, Argonautes and RNA-dependent RNA polymerases. Previous biochemical studies have also suggested that Qip, with an exonuclease domain, facilitates the conversion of duplex small interfering RNAs into single strands. In our study, the *Qip* gene in *Fusarium oxysporum* was disrupted using homologous recombination technology. The deletion of the *Qip* gene resulted in a decrease in colony growth rates but increased the number of branches. Additionally, the Δ*Qip* mutant had a reduced pathogenicity in cabbage. Our results show *Qip* gene in *F. oxysporum* is required for normal hyphae morphology and virulence. The mutant will be useful for elucidating the relationship between the RNA-silencing mechanism and hyphal growth and development in *F. oxysporum*.

## 1. Introduction

Fungal small ribonucleic acids (RNAs) of 20–40 nucleotides (nt) are involved in RNA-silencing, which can regulate the expression of target genes and therefore are involved in a variety of biological processes, such as development, antiviral defence and the maintenance of genomic stability (Brennecke et al. 2003; Sijen and Plasterk 2003; Baulcombe 2004; Lu et al. 2005). Based on their RNA precursors and biogenesis mechanisms, these small RNAs can be divided into various types in fungi, including QDE-2-interacting small RNAs (qiRNAs), microRNA-like RNAs (milRNAs), Dicer-independent small interfering RNAs (disiRNAs), small interfering RNAs (siRNAs), long terminal repeat retrotransposon-siRNAs (LTR-siRNAs) and tRNA-derived RNA fragments (tRFs) (Jöchl et al. 2008; Lee et al. 2009; Nicolas et al. 2010; Nunes et al. 2011). The maturation of small RNAs in the RNA-silencing pathway is dependent on the RNase III ribonuclease Dicer, RNA-dependent RNA polymerases (RdRps) and Argonaute proteins (Lee et al. 2010). Long double-stranded RNA is cleaved into siRNAs by Dicer (Carmell and Hannon 2004). Single-stranded siRNAs guide the RNA-induced silencing complex (RISC), which includes the Argonaute family of proteins, to combine with endogenous messenger RNA and mediate gene silencing (Martinez et al. 2002). Some forms of aberrant RNA are converted into dsRNA by RdRP, which is hypothesized to trigger the initiation of the pathway (Shiu and Metzenberg 2002).

The Qip exonuclease, first identified as a QDE-2 (an Argonaute homologue)-interacting protein in *Neurospora crassa*, converts duplex siRNA into single strands to activate the RISC (Maiti et al. 2007). *Qip* is required for the RNA-silencing machinery in quelling, which is vegetative-specific, meiotic silencing by unpaired DNA (MSUD) and normal sexual development (Lee et al. 2010; Xiao et al. 2010). Qip, together with QDE-2 and exosome, mediates the maturation of milR-1 milRNAs (Xue et al. 2012). However, the possible participation of the RNA-silencing machinery and mutant phenotypes modulated by distinct classes of endogenous small RNAs (esRNAs) in this process in *Fusariumoxysporum* is still unknown.

The filamentous fungus *F. oxysporum* is an important soil-borne phytopathogen that causes vascular wilt in many economic crops, such as cabbage, tomato, banana and watermelon, worldwide (Armstrong and Armstrong 1981), resulting in incalculable agricultural and economic losses. For effective blight control, research on *F. oxysporum*’s physiological and pathogenic mechanisms is urgent. The completion of genome sequencing provides more information for gene functional studies (Thatcher et al. 2012; Schmidt et al. 2013). In this study, we report the phenotype of a Δ*Qip* mutant of *F. oxysporum*.

## 2. Materials and methods

### 2.1. Strains, media and growth conditions

*F. oxysporum* f. sp. *conglutinans* wild-type strain A8 (race 1), which is native to Italy, is now maintained at the Beijing Academy of Agriculture and Forestry Science. Microconidial suspensions were stored in glycerol at −80°C until used. This strain was used for the preparation of protoplasts in the targeted gene knockout experiments. The *Escherichia coli* DH5a strain used for standard cloning steps was grown in Luria–Bertani (LB) medium at 37°C (Hooykaas et al. 1977) supplemented with ammonia benzyl and agar for a solid medium. Potato dextrose agar (PDA, consisting of 20% potato, 2% dextrose and 1.8% agar) containing 200 μg/ml hygromycin B (Roche, Branchburg, NJ, USA) or 200 μg/ml neomycin (Amresco, Solon, OH, USA) was used to select hygromycin-resistant and neomycin-resistant transformants, respectively (Liang et al. 2014). To maintain their phenotypes, transformants were cultured on PDA plates with 100 μg/ml hygromycin B. The wild-type A8 strain and the *Qip* deletion mutants (Δ*Qip*) were cultured at 28°C.

### 2.2. Isolation of the *Qip* homologue from *F. oxysporum* A8

To isolate the *Qip* gene fragment together with flanking regions from *F. oxysporum* f. sp. *conglutinans* wild-type strain A8 (race 1), the primer pairs Qip-F/R, Qip-Up-1F/1R and Qip-Down-1F/1R (Table 1) were designed based on the high sequence similarity to the *F. oxysporum* f. sp. *lycopersici* strain’s genome sequence and used for polymerase chain reaction (PCR) amplification with genomic DNA from wild-type A8 as the template. PCR products were sequenced by ABI 3730 sequencer (SunBio Company, Beijing, China). A BLAST program was used to search for homologous DNA, and the DNAMAN program was used for amino acid sequences. Protein sequence comparisons were performed using MEGA 5 (Tamura et al. 2011).

**Table 1.** Primer sequences and amplicon lengths.

Prime name	Prime sequence (5′–3′)^a^	Amplicon length (bp)
Qip-F	GACAACAACTACGGTGGG	1319
Qip-R	TTCCGAATGTCTTCCAGT	1351
Qip-Up-1F	GGGACTAGTCAGACCCTTACAACGC	
Qip-Up-1R	ATGGAATTCTTCAACTGGAGGAGCA	
Qip-Down-1F	CCCAAGCTTGTCTTTAGGACCCTTTATT	1238
Qip-Down-1R	AGGGGTACCTCTTTGGTAAGTTGAGCA	
Qip-Up-2F	GAAGAGGAGAAGGGCGACATTAG	947
Qip-Up-2R	GCTCACCGCCTGGACGACTAAAC	
Qip-Down-2F	GTCCGAGGGCAAAGGAATAGAGT	990
Qip-Down-2R	TTGCCTGGGTGGTGGTCTGAAAG	
Qip-Up-3F	CTTTCGTCCCTTGTATGG	1776
Qip-Up-3R	ATGTCCTCGTTCCTGTCT	
Qip-Down-3F	TCTGGACCGATGGCTGTG	1392
Qip-Down-3R	GCGAGGGTTACGCTTCA	
Qip-1F	TCCGCTGGCTCACCAATCTA	1165
Qip-1R	GCAATACCAAGCATGGCACA	

Note: ^a^Restriction sites are underlined.

### 2.3. Generation of the *Qip* gene disruption cassette and mutant

**Figure 1.**
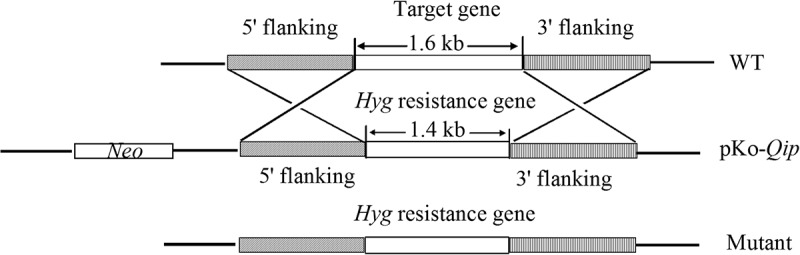
Knockout strategy for the *Qip* gene in *Fusarium oxysporum.*

To construct the *Qip* gene disruption cassette, 1.3-kb upstream and 1.2-kb downstream sequences of the *Qip* gene were amplified with the primer pairs Qip-Up-1F/1R and Qip-Down-1F/1R (Table 1), respectively. The PCR products, containing *Eco*RI-*Spe*I and *Hin*dIII-*Kpn*I restriction sites, respectively, were cloned into pKOV21 using the same sites. The primer pairs Qip-Up-2F/2R and Qip-Down-2F/2R were used to amplify two 0.9-kb fragments, which were used to test for homologous recombination. Wild-type A8 strain protoplasts were prepared as described previously (Talbot et al. 1993). Protoplasts were transformed with the knockout plasmid pKO-Qip using a PEG-CaCl_2_-mediated procedure (Nakayashiki et al. 1999). A schematic diagram of the gene disruption strategy in *F. oxysporum* is shown in Figure 1. Transformants could grow on the PDA plates supplemented with hygromycin B (200 μg/ml), but could not grow on PDA plates supplemented with neomycin (200 μg/ml) represented putative mutants.

### 2.4. Identification of the *Qip* deletion mutant

The putative mutants were analysed further by PCR. The primer pairs Qip-Up-3F/3R and Qip-Down-3F/3R were used to test for homologous recombination at the upstream and downstream flanking sequences. Primer pair Qip-F/R (Table 1) amplified a 1.1-kb fragment in the wild-type but not in the *Qip* deletion mutant. Additionally, genomic DNAs from the wild-type A8 strain and *Qip* deletion mutants were extracted by the method of CTAB. The hygromycin resistance gene’s copy numbers were analysed by genomic Southern blots using a digoxigenin labelling and detection system (High Prime DNA Labeling and Detection Starter Kit I, Roche Applied Science, Beijing, China) according to the standard protocol of the manufacturer.

### 2.5. Identification of mutant phenotypes

To analyse phenotypes, fresh conidia were collected from potato dextrose broth (PDB), washed in sterile distilled water and finally resuspended in sterile distilled water to a concentration of 1 × 10^5^ conidia/ml.

#### 2.5.1. Colony morphology and margins of wild-type strain A8 and the Qip deletion mutant

For morphological analyses of the wild-type strain A8 and the *Qip* deletion mutant, 10 µl (1 × 10^5^ conidia/ml) of the suspension was dropped onto the centre of PDA plates and cultured at 28°C for 5 days. Additionally, 1 µl (1 × 10^5^ conidia/ml) of the suspension was cultured on PDA plates at 28°C for 36 h. The colony margin of wild-type strain A8 and the *Qip* deletion mutant were observed using a Zeiss Axiostar Plus microscope (Oberkochen, Germany).

#### 2.5.2. Microscopic examination of mycelial morphology and conidial morphology

Conidial suspensions from wild-type strain A8 and the *Qip* deletion mutant strain were examined using an Olympus BX-51 microscope. Fresh conidia were inoculated on PDB medium at a concentration of 1 × 10^5^ conidia/ml and incubated on a shaker at 28°C for 72 h. Then, 5 µl of the culture was placed on glass slides for microscopic observation.

### 2.6. Pathogenic measurements

#### 2.6.1. Plants and growth conditions

Disease-sensitive cabbage seeds were covered with sterile gauze and then hydrated in sterile distilled water at ~37°C for 60 min. After treatments, seeds were incubated at 28°C for 1 day until germination.

#### 2.6.2. Pathogenicity assays

The root-dip inoculation method was used to assess the virulence of the wild-type A8 strain and its *Qip* mutant on cabbage in this experiment (Ospina-Giraldo et al. 2003).

## 3. Results and discussion

### 3.1. Identification of *Qip* from *F. oxysporum*

**Figure 2.**
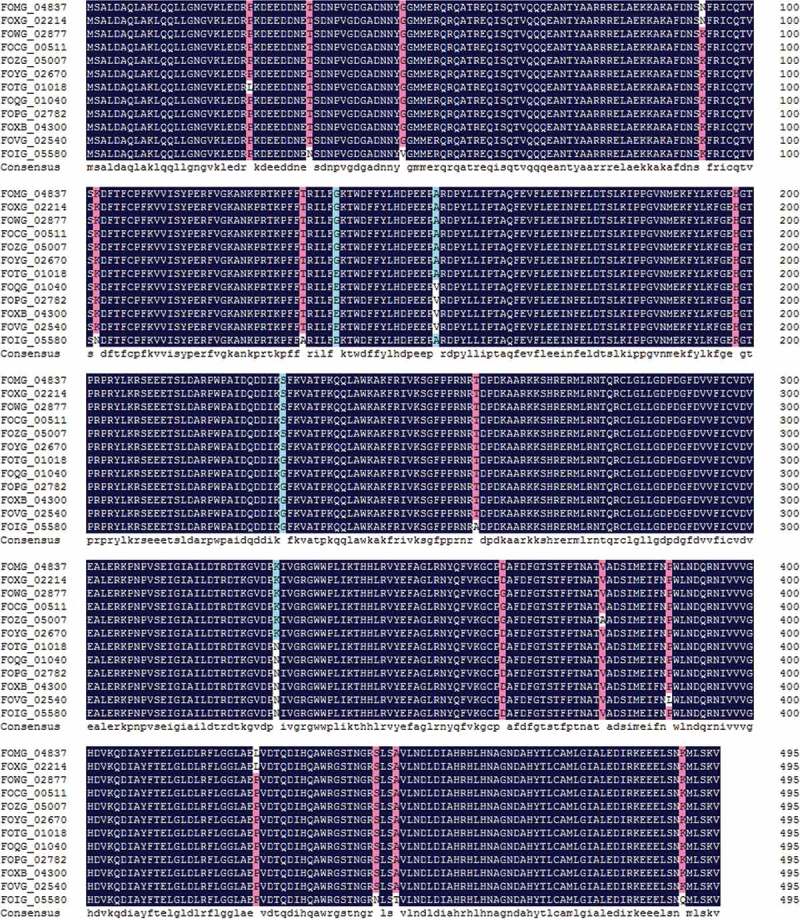
Multiple sequence alignment of putative Qip proteins in *Fusarium oxysporum.*

To identify *Qip* genes in *F. oxysporum*, we first downloaded all annotated protein sequences of *F. oxysporum* from NCBI (http://www.broadinstitute.org/annotation/genome/fusarium_group/MultiHome.html), and a total of 12 annotated protein sets derived from 12 *F. oxysporum* strains were obtained. We used the Qip (QDE-2) protein of *N. crassa* (id: ABQ45366) as the query to search against the annotated proteins of *F. oxysporum*. Interestingly, each of the 12 *F. oxysporum* strains had only one protein with a conserved DEDDh domain near the N-terminus (aa 26–122), which indicates they belong to the 3′-5′ exonuclease domain family, and could be Qip homologues. We further analysed the sequence features of these 12 putative *F. oxysporum* Qip proteins using a multiple sequence alignment. The amino acid sequences of *F. oxysporum* Qip protein were conserved, with all 12 being 495 amino acids in length (Figure 2). Sequence diversity was detected in only 15 loci, suggesting that the functions of these proteins are also conserved. Based on the high sequence similarity among putative *F. oxysporumQip* genes, we designed primer pairs to amplify the *Qip* gene in *F. oxysporum* f. sp. *conglutinans* wild-type strain A8 (race 1). As a result, we successfully amplified the *Qip* gene.

### 3.2. The *Qip* gene was disrupted in the mutant Δ*Qip*

**Figure 3.**
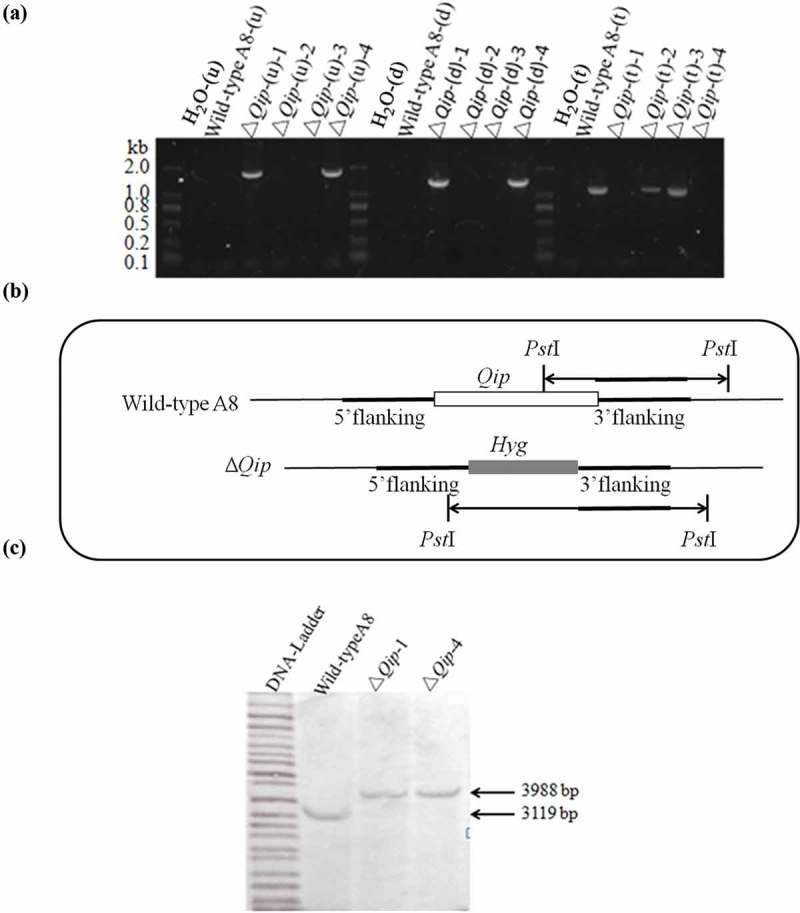
Molecular verification of the *Qip* deletion mutant in *Fusarium oxysporum*. (a) PCR analysis of transformants. (b) Enzyme used for Southern blotting. (c) Southern blot of the *Qip* gene deletion mutant.

Hygromycin-resistant, neomycin-sensitive transformants were isolated for PCR testing. PCR using the primer pairs Qip-Up-3F/3R and Qip-Down-3F/3R were used to produce 1.7-kb and 1.3-kb fragments, respectively, and the Qip-1F/1R primers were used to detect the target gene, confirming the mutant was generated by homologous recombination (Figure 3(a)). Genomic DNA from the Qip deletion mutant and wild-type A8 was digested with the *Pst*I restriction enzyme. Southern blots of the digested products were hybridized using the 1238-bp PCR product as the probe (Figure 3(b)). The hybridization bands of the wild-type A8 and Qip deletion mutant were consistent with the expected DNA fragments (3.9 kb and 3.1 kb, respectively, for digestion with *Pst*I). Therefore, the genome of the Δ*Qip* mutant contained only one copy of the hygromycin-resistant gene (Figure 3(c)).

### 3.3. *Qip* gene is not required for conidial morphology but quantitatively affects hyphal growth

**Figure 4.**
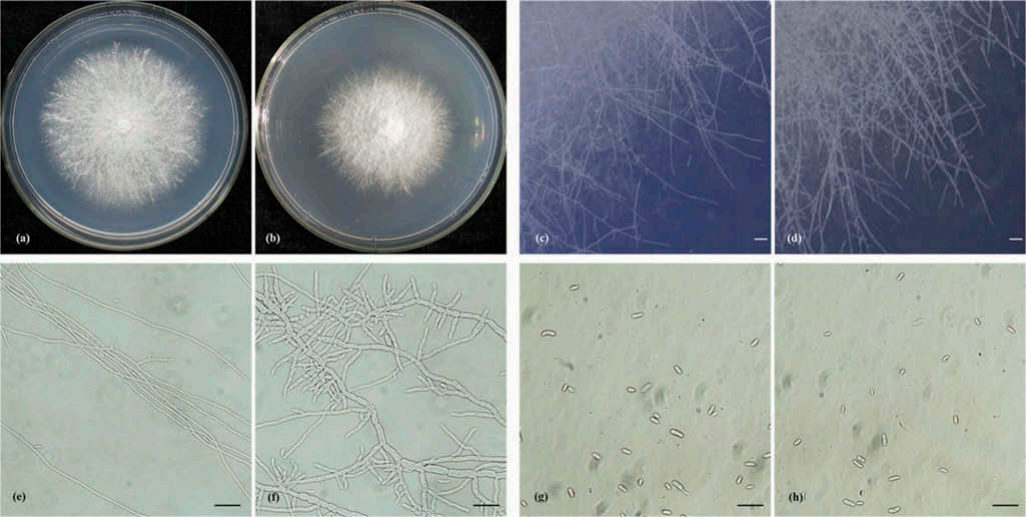
Phenotypic comparison between wild-type *Fusarium oxysporum* strain A8 and its Δ*Qip* mutant; (a), (c), (e), (g) wild-type *Fusarium oxysporum* strain A8; (b), (d), (f), (h) Δ*Qip* mutant; (a), (b) colonial morphology of the wild-type A8 and Δ*Qip* mutant; (c), (d) colonial margin of the wild-type A8 and Δ*Qip* mutant; (e), (f) mycelial morphology of the wild-type A8 and Δ*Qip* mutant; (g), (h) conidial morphology of the wild-type A8 and Δ*Qip* mutant. Scale bars: 1 cm ((c) to (h)).

Colonies formed by the Δ*Qip* mutant on PDA plates had slow growth rates (Figure 4). Additionally, the Δ*Qip* mutant’s colony edges were phenotypically distinct from those of the wild-type A8, forming relatively compact colonies with more branching (Figure 4). We monitored the growth of the conidiogenic hyphae and noticed that there was no difference in the hyphal morphology between the wild-type and the *Qip* mutant in the first 16 h. After 16 h, the Δ*Qip* mutant hyphae became more branched than those of the wild-type A8 (Figure 4). The conidial morphology was similar to that of the wild-type A8 (Figure 4). Normal filamentous fungi grow by the delivery of the components for cell wall extension to the hyphal tips (Girbardt 1957; Riquelme et al. 1998). Hyphal branching is generated when the biosynthetic capacity or volume of the cytoplasm in a hyphal tip has reached its maximum (Yarden et al. 1992). Therefore, in the Δ*Qip* mutant, the cessation of hyphal tip elongation generates a signal to form hyphal branches. Numerous mutants have a defect in hyphal tip growth and branching (Perkins et al. 2000), including mutants in signalling pathways (Lee et al. 1998), the cytoskeleton (Xiang and Morris 1999), subunit of the Vacuolar H^+^ (Bowman et al. 2000) and protein kinases (Wang et al. 2011). This result suggests that the *Qip* gene, a component of the RNA-silencing pathway, was active in other pathways to regulate normal hyphal growth and development. Other RNA-silencing genes, such as Dicer, Argonaute and RdRP, also have similar functions. *Dcl-1* mutants accumulated two size classes of siRNAs and displayed reduced growth rates and altered hyphal growth in *Mucor circinelloides* (Nicolás et al. 2007). Deletion of the CaDCR1 gene in *Candida albicans* results in a severe slow growth phenotype (Bernstein et al. 2012). In *M. circinelloides,* Ago-1 was involved in the response to environmental signals in vegetative development (Cervantes et al. 2013). Two *Dcr* genes are involved in vegetative growth, and the *dcr2* and *rdr3* genes control reproductive development in *Trichoderma atroviride* (Carreras-Villaseñor et al. 2013). These data indicated that the *Qip* gene, together with other RNA-silencing genes, regulates the process of normal hyphal growth and development.

### 3.4. *Qip* is strictly required for the pathogenicity of *F. oxysporum* in cabbage

**Figure 5.**
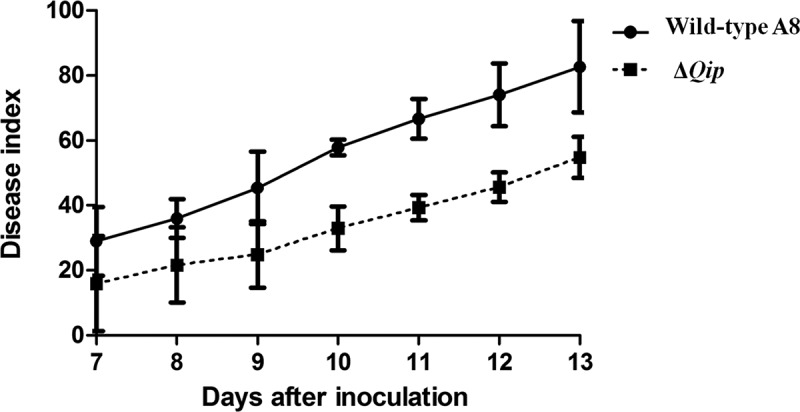
Pathogenicity of wild-type *Fusarium oxysporum* strain A8 and the Δ*Qip* mutant.

To assess the involvement of *Qip* in *F. oxysporum*’s pathogenicity in cabbage, root infection assays were performed. The *Qip* knockout mutant had a reduced pathogenicity phenotype, significantly different from that of the wild-type A8 in disease-causing ability (Figure 5). The result confirmed that growth impairment appeared to be significant in pathogenicity. However, we have not clearly shown that the reduced pathogenicity is due to the Δ*Qip* mutation, its reduced parasitic ability or the decrease in pathogenic metabolites. Previous studies showed that a range of pathogenic factors were required for the pathogenicity of *F. oxysporum*. Mitogen-activated protein kinase (MAPK) and cAMP protein kinase A (cAMP-PKA) signal transduction systems control important steps during plant infection. Some MAPK genes, such as *Fmk1*, Fga1 and Fgb1, of *F. oxysporum* play significant roles in the pathogenicity (Di Pietro et al. 2001; Jain et al. 2002, 2003). RNA-silencing pathways regulate the MAPK and the cAMP-PKA cascades to affect virulence in *F. oxysporum* at the stage of infection.

Since *F. oxysporum* directly penetrates through the roots without appressorium (Lagopodi et al. 2002), the most probable way to gain access to and colonize its hosts is by using its cell wall–degrading enzymes (Ospina-Giraldo et al. 2003). The *SNF1* gene, encoding a cell wall–degrading enzyme, was also involved in the process of pathogenicity. Thus, in the Δ*Qip* mutant, the slow growth may be a result of the reduced production of these enzymes, which probably impedes the initial penetration into the host root system. In other cases, the absence of RNAi allows increased transposition activity, which has been suggested as a mechanism to increase virulence (Wang et al. 2010).

## 4. Conclusion

The RNA-silencing mechanism regulates eukaryotic development and organization. Though several classes of endogenous small RNAs have been identified in Δ*Qip* mutants of *N. crassa*, their biological functions still remain unknown. To clarify the function of the *Qip* gene in *F. oxysporum*, we first constructed a *Qip* gene knockout mutant, using homologous recombination, for a deeper analysis of the transcriptome sequencing and the changes in esRNAs. Our results showed that the *Qip* mutant has a significantly reduced growth rate and that the hyphal morphology was smaller with more branching than that of the wild-type A8. The Δ*Qip* mutant also demonstrated reduced virulence on cabbage plants. These results indicate a role for this component of the RNAi machinery in the control of hyphal development and pathogenicity in *F. oxysporum.*

## Disclosure statement

No potential conflict of interest was reported by the authors.
